# Condoms and sexual health education as evidence: impact of criminalization of in-call venues and managers on migrant sex workers access to HIV/STI prevention in a Canadian setting

**DOI:** 10.1186/s12914-016-0104-0

**Published:** 2016-11-17

**Authors:** S. Anderson, K. Shannon, J. Li, Y. Lee, J. Chettiar, S. Goldenberg, A. Krüsi

**Affiliations:** 1 Gender and Sexual Health Initiative, British Columbia Centre for Excellence in HIV/AIDS, St. Paul’s Hospital, 608-1081 Burrard Street, Vancouver, BC V6Z 1Y6 Canada; 2 Faculty of Medicine, Univeristy of British Columbia, Vancouver, BC V6T 1Z3 Canada

**Keywords:** Migrant sex workers, Criminalization, Third party actors, HIV/AIDS, Sexual health

## Abstract

**Background:**

Despite a large body of evidence globally demonstrating that the criminalization of sex workers increases HIV/STI risks, we know far less about the impact of criminalization and policing of managers and in-call establishments on HIV/STI prevention among sex workers, and even less so among migrant sex workers.

**Methods:**

Analysis draws on ethnographic fieldwork and 46 qualitative interviews with migrant sex workers, managers and business owners of in-call sex work venues in Metro Vancouver, Canada.

**Results:**

The criminalization of in-call venues and third parties explicitly limits sex workers’ access to HIV/STI prevention, including manager restrictions on condoms and limited onsite access to sexual health information and HIV/STI testing. With limited labour protections and socio-cultural barriers, criminalization and policing undermine the health and human rights of migrant sex workers working in –call venues.

**Conclusions:**

This research supports growing evidence-based calls for decriminalization of sex work, including the removal of criminal sanctions targeting third parties and in-call venues, alongside programs and policies that better protect the working conditions of migrant sex workers as critical to HIV/STI prevention and human rights.

## Background

Like other service industries, the sex industry includes a variety of ‘third party actors’, such as receptionists, managers, advertisers, website providers, drivers, housekeepers and security guards, who are involved in commercial sex transactions in roles other than direct sellers (e.g. sex workers) or buyers (e.g. clients). A growing body of research indicates that the services of ‘third party actors’ in in-call indoor sex work venues, such as owners, managers, and receptionists, can critically shape health and safety outcomes [1–6]. Emerging qualitative and epidemiological research reveals, for example, that managerial support of condom use and sexual health education in sex work venues is a significant predictor of HIV risk reduction [7–13].

The criminalization of some or all aspects of commercial sex transactions, including third party actors, is the dominant legislative approach worldwide. Growing evidence globally has demonstrated that criminalization and enforcement-based approaches targeting sex workers negatively impact sex workers’ health, safety and human rights, including risks for violence, poor sexual health, and HIV/STI infection [14–19]. Research suggests that when punitive laws criminalize some or all aspects of sex work [15, 16, 18], police have broad latitude to arrest or threaten arrest of sex workers. In criminalized contexts, research links indirect consequences of policing (e.g. fear and distrust of police, displacement to isolated spaces) and direct harassment and abuse by police (e.g. involuntary detainment, physical abuse, sexual violence) to the reduced ability of sex workers to screen prospective clients or negotiate terms of sexual transactions, including condom use, types of services and fees [6, 16, 20, 21]. Criminalization of sex workers has also been shown to engender increased workplace and societal stigma, including discrimination by healthcare providers, which is associated with reduced access to health services, such as HIV/STI testing and treatment [22, 23]. Recent reports from Human Rights Watch and Open Society Foundations have documented police use of condoms held by sex workers as evidence to enforce sex work laws. This highlights the need for greater attention to the impact of policing on sex worker’s access to condoms [24, 25].

While most of the research to date has focused on the criminalization of sex workers themselves a few studies have recently documented how the criminalization of sex buyers [14] and third parties [3] reproduces the negative effects of the broader criminalization on sex work. This research is of particular importance given efforts across the global north and south aimed at adopting the “Nordic model” of criminalizing sex buyers and third parties, while leaving the selling of sex legal, in an attempt to protect women. However, there is very limited research on the health consequences of this approach, particularly on how policing and criminalizing third-party actors impacts managerial practices and HIV/STI prevention among indoor sex workers, including access to condoms, sexual health training and health outreach services. There is a dearth of research on the impact of laws and policing targeting sex work businesses and third party actors on health and safety of workers, outside of critical work by Bruckert and Tu [3].

Furthermore, there is limited research on the experiences of migrant sex workers in Canada, despite explicit concerns that this group of sex workers face significant barriers to accessing police and legal protections [26]. Research among migrant sex workers globally suggests that this group may face significant socio-structural pressures that negatively impact health and safety risks, including social isolation, cultural and language barriers, insecure immigration, migrant or refugee status, and fear of authorities [26–30]. Studies from diverse settings including South Africa, India and the UK have shown decreased contact with healthcare providers and reduced control over work environments due to immigration, language and socio-cultural barriers [31–33], while other studies have shown that migrant sex workers experience increased economic and social opportunities, and higher mobility that may buffer HIV/STI risks [34, 35]. Heterogeneity among migrant sex workers’ experiences and health outcomes warrants greater attention to the socio-structural factors and legal environment that shape labour conditions and health and safety risks.

This study is located in the Greater Vancouver Area of Canada, which includes 22 urban municipalities and a population of two million people. Fifty percent of residents are immigrants to Canada [36], with Asian immigrants constituting over 65% of new immigrants [37]. The stigma and criminalization of sex work makes it very difficult to determine the exact number of indoor sex workers or sex work venues in the region, however, estimates indicate that there are hundreds of licensed in-call venues and unlicensed micro-brothels in Greater Vancouver [38]. Micro-brothels are unlicensed in-call sex work venues operated by two or more workers in a rented or privately owned apartment or house. By contrast, in a licensed in-call venue, sex work takes place covertly under the auspices of a legal business, such as a beauty salon, an acupressure clinic, massage parlour or body rub studio.

Although sex work laws fall under federal jurisdiction, Canadian municipalities actively regulate in-call venues through police raids, city inspections, licensing requirements, fines and license revocations or parlour shut-downs [39, 40]. While the exchange of sexual services was technically legal in Canada when this data was collected, many aspects of sex work were highly criminalized, including the operation of a ‘bawdy house’ (i.e., a physical venue where sex work takes place) and living off of income generated through sex work. The Supreme Court of Canada struck down these laws as unconstitutional in December 2013 for failure to protect the security of sex workers, however, the federal government in December 2014 has implemented new legislation that criminalizes sex buyers, the advertisement of sexual services and third party actors who materially benefit in the context of a commercial enterprise. The data for this study was collected prior to the change in sex work legislation in Canada. Nonetheless, given a push by various jurisdictions globally to criminalize sex buyers and third party actors, there is a pressing need for research on the health and safety impact of sex work laws that criminalize managers and other third party actors who work in in-call sex work establishments.

## Methods

This qualitative study is situated within a larger longitudinal community-based research project investigating the physical, social and policy environments shaping sex workers’ sexual health, violence, HIV/STI risks and access to care in metropolitan Vancouver. The research builds on community partnerships since 2005 with a Community Advisory Board comprised of over 15 community, sex worker and health support agencies. The origins and development of this project and its community partnerships are described in detail elsewhere [41]. The qualitative project runs alongside a longitudinal cohort of over 800 street and off-street sex workers across Vancouver, known as AESHA (An Evaluation of Sex Workers Health Access).

Indoor sex work venue workers, managers and business owners were invited to participate through outreach to in-call sex work venues and online. Eligibility criteria for the current study were: 1) currently working in an in-call sex work establishment within the last 30 days, either as a sex worker, manager/owner, or both; 2) aged 18 years or older; 3) migrant (i.e., born outside of Canada), whether a legal migrant or not. As this qualitative study was situated within a larger ongoing research project with women in sex work (AESHA), sex workers were eligible if they were self-identified women (both cisgender and trans women). Participants were purposively selected to reflect a range of worker and manager/owner experiences.

From June 2013 to December 2014, co-author JL conducted over 430 hours of ethnographic fieldwork within indoor sex work venues to observe physical and social features of the work environment, such as the presence of security guards or cameras, front desk staffing, door locks, postings of price lists or sexual health policies, provision of condoms and availability of personal lockers for workers. All ethnographic observations were conducted within the context of regular weekly AESHA outreach, which included provision of condoms, HIV/STI testing and referrals to social and health supports, regardless of inclusion in study. The co-author JL is also an outreach worker with SWAN (Supporting Women’s Alternatives Network) for migrant sex workers in Vancouver. Observation sessions lasted six hours and JL recorded brief fieldnotes in a research log after each observation outing. The co-authors also include an experiential migrant sex worker and manager (YE) who has both worked in and managed an indoor sex work venue in the Greater Vancouver Area for many years. YE is an alias assumed by the co-author.

Forty six semi-structured interviews were conducted in Mandarin or English between August 2011 and January 2012 by a trained women interviewer and AESHA outreach worker. Interviews were facilitated by an interview guide invoking broad discussions of participants’ experiences in the sex industry, interactions with police, city officials, co-workers, managers and owners, and access to condoms, education, training and outreach services. The interview guide was piloted and revised prior to implementation. Interviews were conducted in a location selected by the participant (usually a private room in their workplace) and lasted between 30 and 120 minutes, were audio-recorded, transcribed and translated into English. The study operated under ethical approval granted by the Providence Healthcare/University of British of Colombia Research Ethics Board and all participants provided informed consent and were remunerated with a CAD $20 honorarium for their time and expertise.

Interview transcripts were coded for themes and emergent categories in Atlas.ti 7 using a detailed codebook generated inductively from the data and through themes identified in related literature. After coding for recurring content themes, quotations related to sexual health and HIV/STI risk were then conceptually categorized in relation to structural determinants of risk and protection, such as policing practices, managerial practices, and migration [42]. Co-authors JL and YE provided input on analysis of transcripts and all narratives are verbatim by participants.

## Results

### Sample characteristics

Of the 46 participants, 23 were sex workers and 23 were managers/owners, of whom 15 were both workers and managers/owners. All participants were migrants of Asian origin (46 Chinese; 1 Thai), and with the exception of one cisgender man who was a manager/owner, all participants identified as cisgender women. Participants had lived in the Greater Vancouver Area for an average of 8.6 years (range of 1 to 12 years) and had a median age of 42 years (interquartile range: 24 to 54). Participants were sampled from five different municipalities, with most working under one of seven different licenses (e.g. Health Enhancement Centre, Body Rub Studio/Salon, Acupuncturist, Acupressure, Beauty Salon, Beauty and Wellness) and a minority (*n* = 3) operating privately in unlicensed venues (i.e., micro-brothels). For an in-depth review of the various licensing regimes refer to our previous analysis on the impact of licensing and policing on safety and violence prevention in indoor sex work venues [39].

### Impact of criminalization and policing on condom access

While condom access plays a critical role in HIV/STI prevention in workplaces, our findings suggest that the criminalization of in-call sex work venues, including police use of condoms as evidence of sex work taking place in a given location, infringes on sex workers’ condom access. Many participants reported that police raid in-call sex work venues and search workers and the premises for condoms.

“When the police came, the parlour was very busy. The workers yelled out. There were clients at the parlour too. The police searched every room and found used condoms. They also questioned all the clients and working women. Women were ID checked and questioned individually. … Finding the used condoms was not a good development for us.” (Participant 32, Worker)

When workers did not use condoms out of fear that police may use them as evidence, workers were at risk of judgment, racism and ridicule by police, as the following narrative illustrates:

“The police went [to the parlour] all the time … twice they saw me inside working … I almost starting crying then, because both times they saw me, and yet they ask me if I work without condoms. They said, “What are you doing? Oh, you are having sex? And you don’t even - I guess you people don’t even use condoms.” (Worker)

Since police identify condoms as evidence of criminal activity, managers/owners of many in-call sex work venues impose limits on workers’ access to condoms, by enforcing rules on storing and disposing of condoms.

I don’t let my workers keep more than 36 condoms in their lockers. … because the biggest package [sold in stores] has 36. If you kept a hundred or so condoms in your locker, you’d be asking for trouble. If you had ten or twenty of them, you would be able to say that they were for personal use. If you had hundreds, it would be harder to explain. …You would be ratting yourself out.” (Worker & Owner)

“Legalization … will result in parlours providing more safety equipment and supplies to working women. For example, we still have to hide any condoms we have on site in case the police find them.” (Worker)

Furthermore, as a result of criminalization and police use of condoms as evidence of sex work, many managers prohibit the delivery of free condoms and other forms of sexual health outreach (e.g., HIV/STI testing) to in-call sex work venues by health outreach workers:

“My boss was afraid of the government inspection because this business was not legal, so they don’t support outsiders to come in and give supplies and do blood testing” (Worker & Owner).

“My last employer refused to have condoms delivered here by outreach programs, and we would have to go and buy some ourselves. At times we didn’t have condoms [onsite], it became frustrating.” (Worker)

While managers and owners in non-criminalized industries are held accountable for ensuring workplace safety, the criminalization of the sex industry exposes in-call sex workplace managers/owners to considerable legal risks if they provide work safety supplies, such as condoms and lubricants.

“I would be breaking the law if I provided [workers in my Body Rub Salon] with condoms. If I gave them condoms, then it would imply that they must rely on selling sex to make money. … [T]he owner is not allowed to give the workers condoms” (Worker and Owner).

Police raids and the use of condoms as evidence are particularly harmful for migrant sex workers, for whom police contact increases vulnerabilities related to immigration status concerns. In addition to fears of arrest, “new immigrants are afraid of their status being revoked or taken away…[or] of not being able to apply for citizenship” (Worker and Owner) as a result of police raids. Fear of deportation and language barriers between workers and police further frustrates workers, who find themselves vulnerable to miscommunication with police and intimidation.

At the same time, workers’ individual access to condoms is also constrained by the broader social stigmatization of sex work, including taboos against the purchase of large number of condoms, which makes it difficult for workers to access adequate quantities on their own.

“…I do like the free condom distribution service, because if I were to go buy condoms in a store, I can’t just buy a few at a time like normal couples, I have to buy a lot, and I worry about what other people would think.” (Worker)

This barrier to accessing condoms is often magnified for migrant workers, due to cultural factors, shame and a desire to avoid drawing negative attention to themselves.

### Impact of criminalization on access to health care & STI testing

In addition to limiting access to condoms, when managers or owners reject health outreach workers due to fear of prosecution or license revocation, their workers also lose access to onsite HIV/STI testing and other medical referrals. Health outreach services are particularly important for HIV/STI care and prevention among in-call sex workers, given the significant barriers sex workers face accessing primary health care and sexual health services, due to occupational stigma [22].

“[In] The last parlour [I worked in], there were nurses who volunteered to do some blood testing for us, but my employer would not let them come in so we didn’t have many opportunities to get our blood drawn. I would always go to my family doctor and ask him to write me a note to do blood work, but I was afraid that my family would see the note so I was afraid to get my blood tested so often.” (Worker)

“[The bosses] really repel this type of [health outreach] service because the business was illegal. They push these [outreach workers] out. Through your help, first of all, we can protect our own health. I don’t have a barrier to ask you where I can go to see a doctor or which doctor I should go to see. We’re mainly receiving health information through your help. …[T]o other people, I can’t talk as straightforward as this, because they don’t do our jobs and they won’t understand. When I saw the [outreach nurse] I don’t have to hold anything back.” (Worker & Owner)

Participants also critiqued collaborations between public health service agencies, such as, the local Health Authorities, who provide the majority of health and prevention services in this setting, and the police, who as outlined by some participants at times entered the in-call venues in tandem to investigate adherence to health & safety licensing requirements and sex work activities. This made managers/owners and workers even more reluctant to allow health outreach workers to offer medical services or condoms in sex work venues:

“X Health Authority used to provide service for these working women, however, they came in one day with police officers. All [the] working women were shocked and afraid. They thought that the X Health Authority had betrayed them and brought police to capture them. So after that incident, most businesses didn’t allow any X Health Authority [personnel] to enter the business premise. They even rejected any services from any other health organizations too.” (Participant 30, Owner)

Alongside vulnerabilities to policing stemming from the criminalization of sex work, language barriers and immigration status, participant narratives revealed that police surveillance of their workplace contributed to a broader sense of mistrust towards outreach services and health workers.

“We don’t want to cause trouble. That’s what Chinese people are used to, living in a foreign country; we very carefully protect ourselves…because if we were to bring ourselves any hassle, our English isn’t good, and we don’t really understand too much about the law. So of course, the further we stay away [from outreach services], the more careful we can be, the better.” (Worker and Owner)

Nevertheless, for migrant sex workers, who also negotiate language, immigration, and cultural barriers to health care, onsite Mandarin-speaking sexual health outreach workers provide a critical opportunity for health care. Many participants either did not know where to access sexual health services or felt uncomfortable speaking with their family doctor about work-related health issues and sexual health testing. Women health outreach nurses accompanied by translators were uniformly described by participants as accessible and non-discriminatory, closing a key gap in sexual health services for in-call migrant sex workers.

“We’re also afraid of letting strangers know about us, and [outreach nurses] who come in already understand our situation, so I wouldn’t feel as anxious inside. I might tell my own family doctor that I have a weird feeling, and so I feel like getting a blood test [for my own certainty], but I still do not want him to find out that I work in the sex trade, so I don’t actively go for blood tests in general.” (Worker)

“…[W]hen a worker has a condom break, then in the period of time after that, she will be especially worried and afraid. If she goes to her family doctor, she wouldn’t know how to raise that concern. But when social workers…come to provide these services to us, we can talk about anything and everything. So I think that we have a need for … for safety and for confidentiality. They’re both very important.” (Worker & Owner)

“…[W]omen working in this trade need certain things more often … you can’t always go see your family doctor for these things. You wouldn’t know what to say to them. Because when it comes to these things, you still want to be secretive. You still want your privacy, and not want anyone to know, even though you really would like some [health care].” (Worker)

For migrant workers, who face additional barriers to accessing sexual health and HIV/STI testing through primary providers due to cultural, Immigration, and familial barriers and stigma of sex work, the lack of access to voluntary and safe testing onsite within the workplace infringes on their health and human rights.

### The impact of criminalization on sexual health training and workplace safety standards

In addition to restricting access to condoms and sexual health services, a criminalized legal environment and prohibitive licensing context incentivizes management to take a ‘hands-off’ approach to sexual health issues, in order to avoid any related negative legal consequences in the case that this could be used as evidence of sex work. This constitutes a crucial missed opportunity as previous work has highlighted the potential role managers/owners can play in sexual health education. As one owner/worker explained, “Since my license does not permit provision of sexual services, I’ve told my workers not to provide that kind of service. If they decided to provide it, that is not my problem” (Owner and Worker). When asked if she provides workers with any information about the health or legal risks of sex work, another Worker/Owner explained:

“[T]hey do their own work and we do our own work. As owners, we do what we are responsible for and we collect our room fees. How they work inside the rooms is their own business. We’re also very clear about the fact that we don’t force them to do it; it’s their own choice to work. They’re doing business directly with the client, and it’s not relevant to the owner.” (Worker and Owner)

“[While conducting raids] police would ask the manager, ‘why don’t you go over and check on them? Do you know what they’re doing inside?’ The manager would say “I don’t know. We only collect the room fee. What they are doing is their own privacy.” (Worker and Owner)

Since ‘living off the avails of prostitution’ or operating a ‘bawdy house’ were criminalized aspects of in-call sex work, many managers/owners took pains to distance themselves from activities and transactions that happen within a worker’s massage room. Consequently, they refrained from providing sexual health training, education or information in the workplace, activities which would produce evidence that police could use to implicate them in the criminalized aspects of their business. Participants emphasized that most of their information about safety practices came from outreach workers or co-workers, not managers or owners. When asked if managers provide workers with information about safer sex practices, one participant explained, “No. We have to know how to protect ourselves.” (Worker) On the other hand, when asked about their thoughts on the legal status of sex work, participants anticipated that within a decriminalized context, owners or workers would have greater freedom to share health information and safety standards for the parlour with workers and clients, for example, by posting agreements or expectations related to condom use in the reception area. As one participant explained,

“…[A]fter legalization we will have to find ways to make our work even safer, to protect the workers and clients’ health. That will definitely make more work for us, but we’re not afraid of the work. As long as we’re happy, making money and having stability, then we will be okay. We don’t want to be stifled, blamed, and losing money [as a result of police raids]” (Worker)

## Discussion

This analysis highlights how particular legal, immigration, and policing structures (e.g. police raids, use of condoms as evidence, and the criminalization of managers) directly shape managerial practices that infringe on migrant sex workers access to HIV/STI prevention and sexual health education. Our findings reveal that police and immigration raids on in-call venues and the criminalization of managers severely restrict migrant sex workers’ access to condoms, health outreach services, HIV/STI testing and sexual health education. Despite considerable interest and enthusiasm in promoting sexual health, participants in this study stressed that managerial provision of condoms, sexual health training or access to onsite HIV/STI testing can expose managers, owners and workers to considerable legal risks under criminalization. The criminalization of third-parties and current enforcement-based approaches targeting in-call sex work venues puts sex workers and managers in the position of having to choose between the competing priorities of safe sexual health practices in the workplace and the avoidance of criminalization through police raids, licensing fines and legal prosecution. For migrant sex workers, the disproportionate targeting by police and immigration and concerns of immigration status, socio-cultural and language barriers compound risks for migrant sex workers.

In the wake of the murder or disappearance of Indigenous and street-based sex workers in Vancouver over the course of three decades [43], researchers, sex worker advocacy groups and the Supreme Court of Canada have stressed the need for safe, accessible indoor sex work venues, as critical to sex workers’ health and human rights [12, 44, 45]. Global research indicates that in-call venues can foster access to condoms, increased ability to screen clients, and access to other onsite support staff who can help negotiate potential problems with clients, including the removal of violent or threatening clients [1, 2, 5, 6, 12]. Encouragingly, municipalities such as the City of Vancouver are updating their licensing regimes to try to address the health and safety needs of sex workers in licensed in-call venues, through a broad consultation process with sex workers, area residents and businesses [46]. However, policy analyses of venue licensing structures reveal, that municipalities may frame in-call venues as legitimate places of work through licensing, while at the same time using police raids, and immigration inspections as a means to regulate, penalize and arrest workers and managers [39, 47]. Our findings suggest that even if municipalities offer accessible licenses for in-call sex work venues, workers will have limited access to condoms and sexual health education so long as police inspections and raids target venue, managers and business owners. The application of criminal law and prohibitive licensing to regulate or eliminate sex work venues displaces sex work to more hidden or isolated work venues, leaving sex workers with reduced access to safer workplaces and without recourse to police support in the case of violence [39]. These risks are particularly compounded for migrant sex workers who face socio-cultural, immigration and language barriers and have reduced access to HIV/STI prevention and sexual health information.

This research lends further support to evidence-based calls for the full decriminalization of sex work, including the removal of criminal sanctions targeting third parties and in-call venues, as critical to improve the sex workers’ working conditions and facilitate HIV/STI prevention. While previous research has documented the harms of criminalization and policing targeting sex workers on HIV/STI prevention [15, 16, 19, 25, 48–52], this study uniquely documents the negative impacts of criminalizing and policing managers and in-call spaces on migrant sex workers’ access to sexual health information and HIV/STI prevention. These findings extend a critical, nascent body of research on the experiences of third-party actors (e.g. managers, owners, bodyguards, drivers, etc.) and their influence on the health, safety and operation of sex work venues [1, 3, 28]. Just as criminalization of clients reproduces harms of violence and HIV/STI risks to sex workers [14], our research indicates that the criminalization of managers diminishes sex workers’ access to sexual health resources and safe working conditions, and as such infringes on health and human rights of sex workers. This study contributes to a growing body of evidence indicating that the criminalization of *any* dimension of sex work transactions (e.g. purchasing, procuring, advertising, or managing sex work) directly compromises the health and safety of sex workers [14–19].

Regretfully, the most recent sex work legislation implemented in Canada in December 2014 continues to prevent sex workers from working with third-party actors such as managers, drivers, and security personnel, due to prohibitions on benefiting commercially from the proceeds of sex work. This is expected to reproduce the barriers to HIV/STI prevention and health care access among sex workers servicing clients in in-call venues. Our findings indicate that laws criminalizing third-party actors generate a legal environment and policing practices that actively undermine workplace health and safety, since management must distance themselves from any actions (e.g. providing condoms, access to health services, education or training, etc.) that could be used as evidence of encouraging an employee to provide sexual services.

The criminalization of third-party actors ostensibly reflects a well-intended impulse to punish or deter any abuse, mistreatment or financial exploitation of sex workers by third parties [53]. Rather than diminish the mistreatment of sex workers, however, our findings reveal that many harmful managerial practices (e.g. restrictions on condoms, sexual health education, and health outreach services) are a result of criminalization and policing practices. In addition to these harms, criminalization leaves sex workers with little capacity to organize for labour rights and no access to police or regulatory bodies if managers or owners breach labour standards or violate workplace health and safety standards [50]. By contrast, in a decriminalized environment, sex workers could more easily access a fuller range of rights and legal provisions against instances of labour, health or safety violations [3]. According to a recent systematic review of research focused on community organizing among sex workers in lower and middle income countries, the ability to organize collectively is essential for sex workers’ health and safety but is often constrained by laws criminalizing sex work [54]. Indeed, in decriminalized contexts such as New Zealand, workplace health and safety standards have been created in consultation with sex workers. This has resulted in safer working conditions and increased capacity to report violence to authorities [55].

Our study also reveals the unique negative health impact of the criminalization of in-call sex work venues among migrant sex workers. Managerial attempts to reduce risks related to the criminalization of sex work, including workplace restrictions on condoms, access to health outreach workers and sexual health education, exacerbate the well documented barriers to healthcare faced by migrant workers, due language, immigration status and cultural stigma around sexual health services and condom access. These findings contribute to existing research documenting the health-related vulnerabilities of migrant sex workers [31, 35], while highlighting the role of structural factors such as, policing and criminalization of sex work, in shaping access to care and health outcomes.

Our findings indicate the critical need for multilingual and migrant sex worker-led outreach health services to in-call sex work venues, in response to the multiple barriers migrant sex workers face in accessing sexual health services. Alongside increased support for existing sex worker-led outreach efforts and sex worker-run support and advocacy organizations [56], we encourage the development of public policy and multilingual programs with and for migrant sex workers that engage, rather than penalize, sex work venue owners and mangers, as pivotal gatekeepers and potential allies in ensuring workplace safety in sex work venues [11].

These findings should be interpreted in light of the following limitations. As noted in our results, participants reported that managers and workers who have had negative experiences with police are more likely to decline interactions with outreach teams, including with sex worker advocacy organizations and researchers. Given this potential self-selection sampling bias, our findings may underestimate the impact of policing and criminalization on access to HIV-prevention and health outreach services within in-call sex work venues. Continued research and outreach efforts are needed to understand and respond to the sexual health needs of sex workers who operate in informal or more hidden venues, such as private homes, hotels and micro-brothels, or independently. Additionally, as outlined above the data presented in this paper was collected in 2013/2014 prior to the change in sex work legislation, which was implemented by the previous Conservative Canadian Government in December 2014. However the findings of this study despite being collected prior to the change in sex work legislation continue to be highly relevant and applicable to the current situation in Canada and globally. While the new Canadian sex work legislation no longer explicitly criminalizes the operation of bawdy houses it states that people who commercially benefit from the sale of sexual services face up to 10 years in prison. As such, the new legislation continues to criminalize third party actors and in this respect does not represent a significant departure from the previous legislation that was deemed unconstitutional by the Supreme Court of Canada for interfering with sex workers’ ability to protect themselves from violence, abuse and HIV/STI infection [57].

## Conclusion

Migrant sex workers’ access to condoms HIV/STI, prevention information and care, including sexual health training, and HIV/STI testing, is a critical human rights and public health issue. Our results highlight how socio-structural factors, namely policing and immigration practices and the criminalization of managers/owners, produce in-call work environments in which sex workers face significant barriers to accessing condoms, sexual health education and services. While all of the sex workers in this study expressed a strong desire to practice safer sex in order to protect themselves from HIV and other STIs, their capacity to do so was severely undermined by police harassment, raids for condoms to use as evidence of criminal activity, and associated managerial fears that sexual health education or access to outreach services would result in criminal prosecution or license revocation. Our findings affirm international evidence-based guidelines by Amnesty International, the World Health Organization, UNAIDS, UNDP, and UNFPA calling for the full decriminalization of sex work, including third parties, as necessary for the health and human rights of sex workers [18].

## Abbreviations

AESHA: An Evaluation of Sex Workers Health Access
HIV: Human immune deficiency syndrome
STI: Sexually transmitted infection
UNDP: United Nations Development Programme
UNFPA: United Nations Population Fund
